# Vigorous Exercise Enhances Verbal Fluency Performance in Healthy Young Adults

**DOI:** 10.3390/brainsci15010096

**Published:** 2025-01-20

**Authors:** Maya M. Khanna, Corey L. Guenther, Joan M. Eckerson, Dion Talamante, Mary Elizabeth Yeh, Megan Forby, Krystal Hopkins, Emmali Munger, Grace Rauh, Shringala Chelluri, Courtney Schmidt, Isabel Walocha, Matthew Sacco

**Affiliations:** 1 Department of Psychological Science, Creighton University, Omaha, NE 68178, USA; coreyguenther@creighton.edu (C.L.G.); tdailoan978@gmail.com (D.T.); maryelizabethyeh@creighton.edu (M.E.Y.); meganforby@creighton.edu (M.F.); krystalhopkins@creighton.edu (K.H.); shringalachelluri@creighton.edu (S.C.); courtneyschmidt@creighton.edu (C.S.); izzywalocha23@gmail.com (I.W.); 2 Department of Exercise Science and Pre-Health Professions, Creighton University, Omaha, NE 68178, USA; joaneckerson@creighton.edu (J.M.E.); emmalimunger@creighton.edu (E.M.); gracerauh@creighton.edu (G.R.); matthewsacco@creighton.edu (M.S.)

**Keywords:** exercise, verbal fluency, lexical features, cognitive performance

## Abstract

Background/Objectives: We examined the effects of cardiovascular exercise on verbal fluency using a between-groups design. Methods: Within our experimental (i.e., exercise) group, participants performed phonemic and semantic verbal fluency tasks (VFTs) before, during, and after a vigorous 30 min bout of cycling. Participants within our control group also completed these VFTs before, during, and after a non-physical activity. We compared the VFT performance of the experimental (exercise) and control (no-exercise) groups of participants in terms of the characteristics of the words that they produced within the VFTs. In addition, we examined these aspects of VFT performance for each participant group across time within the experiment session. Conclusions: From these comparisons, we see that exercise influenced VFT performance. Most notably, participants engaged in exercise changed their VFT performance over time, while control group participants did not. Exercising participants produced more words over the course of their exercise session that contained fewer letters over time and were lower in frequency during and after exercise as compared to before exercise. Additionally, topic switches in the VFTs increased after exercise as compared to before exercise. Participants in the control group did not change their VFT performance over time according to any of these measures. These findings indicate that exercise impacted participants’ lexical access and that these VFT performance changes were not due to practice effects.

## 1. Introduction

Cardiovascular exercise has many well-documented physiological benefits and there is growing evidence that exercise improves cognitive performance, as well. The most well-documented of these cognitive benefits is the retention of cognitive abilities enjoyed by older adults who engage in regular aerobic exercise [1,2]. Much of this research indicates that older adults who routinely exercise perform better than their sedentary counterparts on measures of executive functioning such as planning, decision making, and multitasking [1]. In part, these benefits are believed to be related to structural and functional brain changes that may occur after sustained aerobic training programs [3].

Furthermore, young adults also appear to experience functional connectivity changes when they engage in moderate-to-high levels of cardiorespiratory activity. For example, Ko and colleagues [4] found that healthy young adults engaged in even brief exercise bouts (e.g., 10 min) can exhibit altered functional connectivity within the Default Mode and Attentional Networks as compared to those engaging in relatively low levels of cardiorespiratory exercise [4]. Similarly, high levels of cardiorespiratorytory fitness are associated with increased connectivity between the hippocampus and Default Mode Network areas of healthy young adults [5]. Taken together, the results from Colcombe and Kramer [1,2] examining the impact of sustained aerobic activity program and the results from Kronman et al. [5] and Ko et al. [4] illustrate that both sustained engagement in cardiorespiratory exercise and brief periods of relatively intense physical activity can produce neurophysiological benefits.

Recent research is also examining the potential benefit of acute bouts of exercise (e.g., 20–30 min) for cognition. For example, Ludyga and colleageus [6] conducted a meta-analysis on studies examining the acute effects of moderate exercise (i.e., engaged at 30% to 70% of Heart Rate Reserve (HRR)) on cognitive performance (e.g., executive function tasks). They found that most studies indicate an exercise-related enhancement of reaction time and accuracy in tasks related to executive functioning, and that this was especially the case for studies focused on older adults and preadolescent children. Interestingly, they did not find that the cognitive performance enhancement due to exercise was different for those with higher levels of aerobic fitness compared to those with lower levels of aerobic fitness [6,7].

Others have also investigated the impact of acute bouts of cardiovascular exercise on cognitive processing in children, adolescents, and younger adults [8]. In their review, Verburgh and colleagues [8] found an agreement across studies suggesting that acute bouts of physical exercise were associated with boosts in performance on measures of inhibition, interference, and working memory. In general, the studies that Verburgh and colleagues [8] reviewed found that even a brief bout of exercise (e.g., 10–40 min) was related to improvements in these executive functions. They hypothesized that the results were likely due to a temporary increase in blood flow and cerebral oxygenation within the prefrontal cortex (PFC) that underlie executive functions.

Thus, substantial research has indicated that both long-term and acute exercise can increase cognitive performance in a series of tasks related to executive functioning due to enhanced frontal lobe functioning as well as hippocampal and Default Mode Network functioning, in additional to other neurofunctional areas. However, fewer studies have examined how exercise may influence other behaviors related to frontal lobe functioning, such as verbal processing behaviors. Voss et al. [9] noted that exercise is not only associated with frontal lobe functioning, but also with functional and structural changes within temporal and parietal areas [9]. This pattern of increased functional activity within frontal, temporal, and parietal regions should be associated with increased lexical access and verbal processing [10]. Metzger and colleagues [11] conducted one of the few studies that examined the relationship between exercise performance and verbal performance. The researchers asked participants to complete language production tasks while also measuring the participants’ cerebral blood flow within the frontal, temporal, and parietal areas using functional near-infrared spectroscopy (fNIRS) during a 12 min walking task. Metzger et al. [11] found cerebral blood flow to be increased to both the frontal and temporal areas, especially in Brodman’s areas 44 and 45 (i.e., Broca’s area) during movement. They also found that, when participants were talking while walking, the hemodynamic response was even greater in Broca’s area as compared to walking alone. These findings demonstrate that exercise not only elevates cerebral blood flow in the PFC, but that this hemodynamic response extends to several other cortical regions, including those associated with verbal processing. Thus, this increase in cerebral blood flow during exercise should result in increased verbal processing and lexical access during exercise.

The focus of Metzger et al. [11] was to establish which brain areas receive increased blood flow during motor and motor/verbal dual-task paradigms. However, they did not report changes in verbal behavior itself during exercise. That is, although their findings document elevated blood flow in Broca’s area during exercise, they did not discuss differences in spoken language performance during greater vs. lesser motor exertion. The fact that blood flow increased to Broca’s area during exercise—and even more so if speaking while walking—suggests that verbal processes relying on frontotemporal areas should improve during exercise, as well. For example, tasks engaging lexical access and verbal fluency should benefit from increased activation within frontotemporal areas. Therefore, the present study examined the effects of vigorous exercise on verbal processing.

### 1.1. Assessing Verbal Processing—The Verbal Fluency Task

We believe the verbal fluency task (VFT) is optimal for assessing the impact of exercise on verbal processing because of its association with frontotemporal functioning [12,13,14,15]. The VFT is a classic technique used to assess semantic processing and executive functioning and is frequently used to assess frontal and temporal lobe functioning in both neuropsychological patients and healthy individuals [12,16]. The VFT has both phonemic and semantic versions in which participants generate as many words as they can following a category cue within a 60 sec time frame. In the phonemic version, participants are given a letter, (e.g., F) and asked to generate as many words as possible that start with that letter within 60 s, while, in the semantic version, participants are given a category (e.g., fruit) and asked to generate as many items as possible within the same time frame. Through this simple task, researchers can examine several factors. For example, the number of words that participants generate is used as a measure of lexical access and vocabulary size and the way the words are generated can reveal processing patterns. For instance, the word production pattern and size of clustering (saying a string of words related to one another, e.g., cherry, blueberry, raspberry, strawberry, etc.) indicates the level of temporal lobe (i.e., semantic) processing [12,16,17]. On the other hand, the number of times that a participant switches topics within a string of words (e.g., cherry, blueberry, raspberry, cantaloupe, watermelon, honeydew, kiwi, pineapple, banana, etc.) illustrates task switching, which is a traditional executive function, or frontal-lobe-dependent skill [18].

Finally, we can examine the characteristics of the words participants generate in the VFT. That is, the basic lexical characteristics of the words generated may vary in word frequency, number of letters, and the age at which participants likely acquired the words (i.e., Age of Acquisition, AoA). It is possible that exercise-induced increases in blood flow to frontotemporal regions could influence the characteristics of words accessed by participants, since these characteristics (particularly frequency and AoA) are moderated by frontotemporal lobe activation. For example, Fiebach et al. [19] reported elevated activation in the left inferior frontal and anterior insular regions when participants accessed later versus earlier acquired words. Similarly, Fiebach et al. [20] reported increasing the activation in the left inferior frontal lobe within the pars triangularis and the pars opercularis as the frequency of words produced decreased. Thus, it is reasonable to expect that exercise-related increases in blood flow to the frontotemporal region could influence the pattern of lexical access. Specifically, more unusual words (e.g., lower frequency and more recent AoA values) should be relatively more accessible at times of optimal frontal and temporal lobe functioning such as during high-intensity exercise.

Although the influence of exercise on lexical access has yet to be explored, the influence of exercise on conceptually similar processes has been examined. For example, in a series of studies, Gondola and colleagues [21,22,23] found that exercise enhances expansive thinking, such as creativity and problem-solving. In fact, they found both single-bout and long-term exercise facilitate creativity and divergent thinking. Unsurprisingly, Abraham and colleagues [24] found areas in the left inferior frontal gyrus and temporal pole exhibited elevated activation while participants performed the “alternate uses” task, a widely used measure of divergent thinking. Although these findings do not directly inform whether high-intensity exercise improves access to low-frequency or more recent AoA words, they do demonstrate that exercise facilitates expansive thinking, which could potentially contribute to improved lexical access.

Another advantage of using the VFT to assess lexical processing is that it has been shown by some to be robust against practice effects [25,26]. In fact, Basso et al. [25] found that VFT performance did not increase in participants retested on the VFT, as well as many other cognitive tasks; however, other cognitive measures such as Wisconsin Card Sorting, Ruff Figural Fluency, and Verbal Concept Attainment were prone to practice effects. Furthermore, the most common version of the phonemic and semantic VFTs are designed for multiple administrations within a short period of time, which makes the test well-suited for examining the impact of exercise on cognitive functioning within a single exercise session. However, see Bartels and colleagues [26] and Cooper and colleagues [27] for contrasting views regarding the VFT and practice effects.

The VFT also allows an examination of the pattern of word production, which reveals details about lexical processing and executive functioning. In particular, when people generate words in the VFT, they usually say several words that are related to one another in meaning (e.g., strawberry, cherry, blueberry, etc.) before switching to another series of items that are related to one another (e.g., cantaloupe, watermelon, honeydew, etc.). Troyer et al. [12] reported that the number of words in a thematic cluster is related to temporal lobe processing, while the number of times a participant switches topics is related to frontal lobe processing. In neuropsychological assessments, cluster size and number of switches are used to measure temporal and frontal lobe functioning, respectively, and their potential deterioration [16]. Similarly, Ovanda-Tellez and colleagues [28] argued that switching and clustering behavior in an associative fluency task similar to the VFT can serve as indices of memory and control process functioning and attentional control processes, respectively, again indicating how fluency tasks may serve as a way to gauge frontotemporal processing networks. Perhaps the biggest advantage of using the VFT is that it can be conducted while a person is performing exercise, since it does not require the participant to interact with computer equipment.

### 1.2. Overview of the Present Studies

The present research reports an experiment directly investigating the impact of exercise on verbal fluency by comparing a group of participants engaged in the VFT while vigorously exercising to another group engaged in VFT while engaged in a non-exercise activity. In the exercising group, we examined the impact of moderate-to-vigorous cardiovascular exercise on cognitive functioning by using the semantic and phonemic VFT. We also employed more traditional measures of executive functioning in the form of the Eriksen flanker task [29,30] to measure inhibition and the counting Stroop task [31] to measure attention allocation and inhibition. We compared their performance to a group of participants who were not engaged in exercise. That is, we conducted the same series of tasks on a separate group of participants who were not engaged in cardiovascular exercise, which allowed us to rule out practice effects as an alternative explanation for any exercise-related VFT enhancements observed by our experimental (exercise) group of participants.

From this experiment, we can assess the impact of a brief (i.e., 30 min), but challenging bout of cycling on cognitive functioning and lexical access using the VFT. Our participants exercised at an intensity ranging between 65–75% of their heart rate reserve (HRR). The HRR method accounts for an individual’s resting heart rate (HR) and is recommended for exercise prescription over HR-dependent methods (e.g., a percentage of maximal HR or maximal oxygen consumption), since intensity can be over- or underestimated using those methods. According to the American College of Sports Medicine Guidelines for Exercise Testing and Prescription [32], moderate-intensity exercise is defined as 40–59% HRR, whereas vigorous exercise ranges from 60–89% of HRR. We recruited a total of 95 healthy young adults who habitually exercised (i.e., at least three times per week for at least 30 min each time) for these experiments. We compared the performance of 54 of these participants in the exercise condition to 35 participants in the no-exercise condition. We chose this level of exercise intensity to mirror the level of intensity that was used by the studies that Ludyga and colleagues [6] included in their meta-analysis. In particular, we tried to use a similar cardiorespiratory intensity level that was used by previous studies of physically fit young adults. We also were careful to only recruit participants who were habitual exercisers in both the exercise and control conditions. We wanted to make sure the exercising participants could sustain exertion of 60%+ of their HRR for at least 30 min, and then wanted to ensure that the control group were of a similar level of fitness. We note that it is well-established that regular exercise can improve vascular function, lower blood pressure and resting heart rate, and lead to cardiovascular structural changes within the brain and throughout the body [33]. Thus, we wanted participants in both the exercise and control conditions to have benefitted from habitual exercise in these ways. We felt this would allow a more meaningful comparison in their performance on our verbal processing and executive functioning measures.

Specifically, we compared the performance of the groups on the flanker task, the counting Stroop task, and the phonemic and semantic VFTs before, during, and after their experiment session while the exercise group engaged in vigorous stationary cycling and the control group engaged in a non-exercise task. We hypothesized that participants engaged in exercise would have enhanced performance on the VFT, as well as in executive function tasks as compared to participants not engaged in exercise.

Specifically, we believed that exercise-enhanced verbal processing would lead to broader and deeper lexical access during the VFT, resulting in a greater number of words being produced with different lexical characteristics as compared to words produced when not exercising. In particular, we speculated that cardiovascular exertion would result in an increase in the number of words during the VFT, and that the words produced would be lower in frequency and have more recent AoAs as compared to words produced before exercise. We are able to examine this by first comparing the lexical characteristics of the words produced by participants within the exercise condition, before, during, and after exercise. We are also able to examine the impact of exercise on lexical access by comparing the change in VFT performance over time within the exercising and non-exercising participant groups.

## 2. Method: Experimental (Exercise) vs. Control (Non-Exercise) Groups

### 2.1. Materials and Protocol

Our research protocol was reviewed and approved by the Creighton University Institutional Review Board (Creighton IRB approval code #975634-4-01 assigned 20 December 2018) and conformed to the Declaration of Helsinki. Each participant granted informed consent before participating in experiment sessions. We used a repeated-measures design in which participants completed phonemic and semantic verbal fluency tasks before, during, and after their experiment sessions. Participants also completed measures of attention allocation and inhibition at the beginning and the end of their session.

### 2.2. Participants

**Figure 1:**
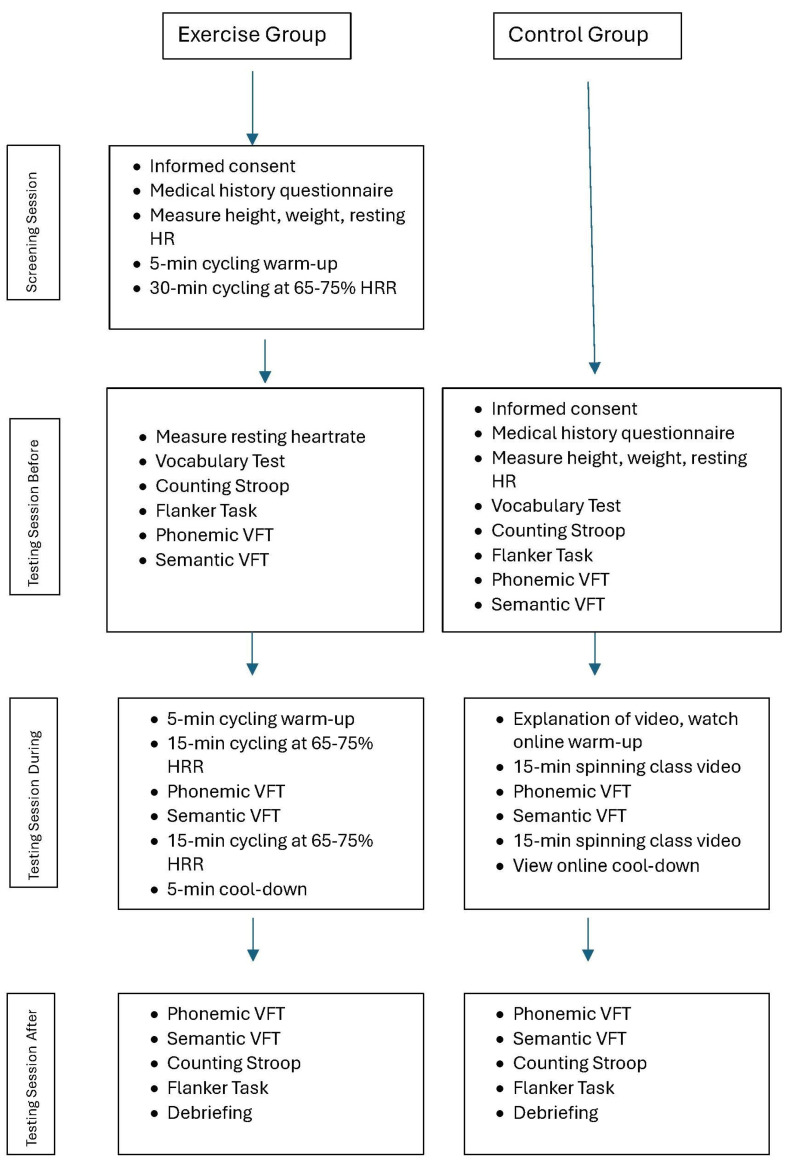
Procedure for experimental (exercise) and control (no exercise) groups; please note that Heart Rate is abbreviated HR and Verbal Fluency Task is abbreviated VFT.

Participants were recruited from Creighton University and included 52 self-identified male and 43 self-identified females. The experimental group included 34 males and 26 females (*M_age_* = 19.33, *SD* = 2.58 years), while the control group included 18 males and 17 females (*M_age_* = 19.03, *SD* = 1.12 years). All participants received partial course research credit in return for their participation. Please see Figure 1 for an overview of the procedure followed for each participant group; in addition, see Table 1 for participant characteristics within each group. To ensure we recruited participants who could complete a vigorous bout of exercise, we recruited participants who indicated on a prescreen survey that they routinely exercised at least three times each week for a minimum of 30 min per session regardless of which participant group they were assigned. We did this so that we could compare the performance of 2 groups of habitual exercisers on the VFT. Recruitment for the experimental (exercise) and control (non-exercise) groups was conducted via two different postings within the online participant recruitment website due to the difference in number of sessions and time of participation for each group. Thus, the assignment into these two groups was not random, but, instead, based on the experiment posting to which a participant responded.

Due to the nature of the exercise conducted with the experimental group compared to the control group, who were not exercising during the experiment, we used a slightly different protocol for experimental (exercise) and control conditions. For the exercise condition, we worked with each participant across 2 sessions—a screening session, and a testing session. The purpose of the screening session was to familiarize exercise participants with the stationary cycling apparatus and to ensure that they had the physical fitness to complete a 30 min bout of vigorous cycling. All testing for the control group was conducted within one testing session. For the sake of clarity, we begin by describing the protocol employed in the exercise condition, and then describe the protocol employed in the control condition.

## 3. Exercise Condition Protocol

### 3.1. Screening Session

In the initial screening/familiarization session, exercise participants completed a medical history questionnaire to screen for health conditions or medications that could interfere with exercise performance (see Supplementary Materials). These exclusion criteria resulted in three participants ending participation in the study. We also measured participant height, weight, and resting HR. We measured resting and exercising HR using a Polar chest-strap HR monitor. The target HR range for exercise was calculated using the HRR method:

Target HR = [(age-adjusted maximum HR−resting HR) × % Intensity] + resting HR

The maximum HR was estimated using the traditional formula 220-age and we selected intensity levels of 65% and 75% to establish an HR range that represented a vigorous level of exercise intensity based upon the ACSM guidelines [32]. For example, a 20-year-old with a resting HR of 75 bpm^−1^ would have a target HR range of 156–169 b‧min^−1^.

Next, participants moved to the cycle ergometer and completed a 5 min incremental warm-up to achieve their target HR, and then completed a 30 min bout of cycling at an intensity ranging between 65–75% of their HRR, followed by a 5 min cool down (or longer if needed) to reach an HR within 20 b∙min^−1^ of their resting HR. Researchers manipulated the workload on an electrically braked cycle ergometer (Corival Lode, Groningen, The Netherlands) during pedaling and monitored the power output (Watts) and HR each minute to ensure the participants maintained the appropriate target HR range. Every 5 min, participants also reported a rating of their perceived effort (RPE) using the Borg scale [34] ranging from 6 (very, very light) to 20 (very, very hard). The screening session was used to familiarize participants with the exercise protocol and to ensure participants were able to complete a 30 min bout of exercise at 65–75% of their HRR. Based on these criteria, two participants chose not to return for the subsequent testing session. At the end of the screening session, each of the continuing participants was scheduled for a testing session within 1 week.

### 3.2. Testing Session

During the testing session, exercise participants were fitted with the chest-strap HR monitor, and resting HR and blood pressure were measured. Next, participants completed a vocabulary measure, the counting Stroop and flanker task to gauge executive functioning, and the phonemic and semantic VFT to gauge verbal processing. The vocabulary measure was completed before exercise only. The executive function tasks were completed both before and after the exercise session. And the VFT was completed before, during, and after the exercise session.

#### 3.2.1. Vocabulary Measure

Participants completed a brief computer-based vocabulary test from the Woodcock–Johnson synonyms test [35] to explore the relationships between vocabulary size and VFT performance and whether this could be influenced by exercise. For this task, participants were presented with one word at a time on the computer screen and asked to provide a synonym (e.g., view the word *street* and produce a synonym such as *road*). Words became progressively more difficult across the 40 items. The participant responses were recorded by one of the investigators within the computer program. We calculated the number of items for which an appropriate synonym was generated and this number divided by the total items (40) served as the vocabulary score (i.e., proportion correct) for each participant.

#### 3.2.2. Executive Function Tasks

Counting Stroop. This task is similar to the classic Stroop task [36]. We chose to conduct the counting Stroop task rather than the classic Stroop task for two reasons. First, our main focus for the experiment was on the verbal fluency task performance. Thus, we wanted the measures of executive functioning to engage verbal processing less than the classic Stroop. Although the classic Stroop asks participants to identify ink color while ignoring the meaning of words presented, it is clear that participants do process the words presented and consider their meaning. We did not want participants to be primed for certain words within the VFTs based on the words presented during a classic Stroop task. We felt that this was less of a concern with the counting Stroop task. Second, we used the counting Stroop as a task that we felt gauged attention and inhibition processes so that we could compare that to the performance on the Eriksen flanker task, which is designed to gauge inhibitory processing. For the counting Stroop, participants complete digit and count versions of the task [31]. In the digit version of the task, participants see a word or words corresponding to a number name (e.g., one) and must indicate the digit of the number presented (i.e., 1). For congruent trials, the number of words presented matches the digit indicated (e.g., one). In incongruent trials, the number of words presented does not match the digit presented (e.g., one one one). For the count task, participants indicate the number of words presented on each trial, while ignoring what the word indicates. For example, in a congruent trial, a participant may see (two two) and indicate two words. For an incongruent trial, the participant may see (two two two) and indicate three words. As with the classic Stroop experiment, performing this task engages attention, since attention allocation abilities are indicated by the differences in reaction time (RT) observed between congruent and incongruent trials. A greater difference in RT between incongruent and congruent trials indicates the greater attentional demands of the incongruent compared to congruent trials. Thus, a decrease in RT difference from congruent to incongruent trials indicates an improvement in attention allocation [31].

Eriksen Flanker Task. This task requires both attention allocation and inhibitory processing [29]. Participants complete a series of trials in which they identify the center letter in a string of letters. The center letter and adjacent letters are either congruent, incongruent, or neutral to one another. For congruent trials, participants see a string of the same letter (e.g., AAAAAAAA), while, for incongruent trials, the center letter and adjacent letters are not the same (e.g., LLLALLL). For neutral trials, non-letter symbols are adjacent to the center letter (e.g., &&&A&&&). Similar to the counting Stroop test, the relative RT difference across the three conditions is believed to indicate the inhibitory capacity of the participant. That is, larger RT differences indicate greater inhibition demands than do smaller RT differences [29,30].

#### 3.2.3. Verbal Fluency Tasks

After completing the counting Stroop and Flanker tasks, participants completed a phonemic trial and a semantic trial of the VFT. For the phonemic task, we used the F-A-S as it has been shown to be robust against practice effects [25]. For the semantic task, we used the animals, fruit, and household objects version of the VFT, once again, due to its robustness against practice effects [25]. In addition, these versions of the VFT have been tested and normed across a wide range of ages [14]. We counterbalanced the order of VFT test presentation across participants; however, for each trial, participants first received the phonemic version of the task followed by the semantic version.

For the VFT, participants were given standard instructions. That is, for the phonemic VFT, participants were asked to name aloud as many words as they could starting with a given letter (e.g., F) within 1 min, but not to provide proper names. For the semantic VFT, participants were given a category (e.g., animals) and were asked to name as many members of that category as possible within 1 min. During the VFT, a researcher wrote down the verbal responses and also recorded the responses on a digital audio tape recorder and transcribed the audio recording of the words to verify that the written and audio recordings matched. After the first pair of VFTs, participants then moved on to the cycling portion of the testing session.

#### 3.2.4. Cycling Task

For the testing session, participants repeated the cycling procedure conducted during the screening session. That is, they completed a 5 min warm-up, followed by a 30 min bout of vigorous cycling. A researcher adjusted the ergometer workload to maintain a power output to keep the participant’s HR between 65% and 75% of his/her HRR. The power output and HR were recorded every minute and participants were asked to report their RPE every 5 min. Fifteen minutes into cycling, participants completed a phonemic and a semantic VFT. They carried this out while cycling and while maintaining their power output and target HR; once more, the VFT only requires a verbal response, and no interaction with a computer, so participants were able to complete it while cycling. A researcher created a written and audio recording of participant responses. After these two VFTs, participants completed the remaining 15 min of cycling and then entered a cool down phase for at least 5 min to achieve an HR that was within 20 b·min^−1^ of their resting HR.

#### 3.2.5. Post-Cycling Assessments

Once participants completed the cycling task, they then performed a final series of phonemic and semantic VFTs, followed by another administration of the counting Stroop and flanker task.

## 4. Control Condition Protocol

Control participants began the testing session by completing a consent form and the medical history questionnaire (see Supplementary Materials). Next, just as those in the exercise condition did, control participants completed the Woodcock–Johnson synonyms vocabulary task, an initial administration of the counting Stroop and Eriksen flanker tasks, and the same pre-task phonemic and semantic trials of the VFT. They then moved on to the testing phase of protocol. Whereas, at this point, exercise participants actually engaged in vigorous cycling for 30 min, control participants merely watched others engage in vigorous cycling for the same period of time. More specifically, control participants watched a video of a spinning class (i.e., vigorous stationary cycling) online for 30 min. We chose to use a spinning video in order to have participants in the exercise and control groups exposed to similar dialogue (i.e., cycling-related dialogue) and similar stimuli during the study. Because participants in the control group were not engaged in exercise, we did not ask them to indicate their RPE throughout the session as we did with participants in the exercise condition. After initially completing the Woodcock–Johnson test, the counting Stroop, the Eriksen Flanker task, and their first set of VFTs, participants were directed to watch a video of a guided spinning class (see video at—https://www.youtube.com/watch?v=Pvg9c0-iYgg). Participants were asked to watch this video without distraction for 15 min. After 15 min of the online spinning video, a research assistant paused the video and completed the second set of phonemic and semantic VFTs with the participant.

After the middle set of VFTs, the research assistant continued the spinning class video where it had been paused. Participants then continued to watch the spinning video for another 15 min. After the time elapsed, the research assistant conducted the last set of phonemic and semantic VFTs. Then, participants completed a second round of counting Stroop and flanker tasks. Finally, the participant’s ending HR was measured and recorded.

Thus, control participants’ experience and the timing of our primary assessments mirrored that of exercise participants’ in all ways, with the exception of the cycling task—whereas exercise participants actually engaged in vigorous cycling during the testing phase, control participants merely watched others engage in vigorous cycling.

## 5. Results

There was a substantial amount of data recorded related to both our physiological and cognitive measures. We will report descriptive information related to the physiological data (see Table 1), but will report both descriptive and inferential analyses related to measures of cognitive performance. Our focus in data analyses was on the information related to the VFT performance, as this level of lexical feature analyses of VFT performance is novel.

### 5.1. Vocabulary

We measured participant vocabulary using the Woodcock–Johnson synonyms task and found that, within the experimental group, there was an average accuracy for the task of 73.2% with a range of 45–83% correct and a standard deviation of 6.25%. In the experimental group, we found no relationship between the vocabulary score and VFT performance in terms of the number of words produced during the VFT (all rs < 0.029); however, there was a trend towards a relationship between the vocabulary score and semantic VFT before exercise, *r*(51) = 0.238, *p* = 0.092. For the control group, there was an average accuracy of 72.7% with a range of 59–86% and a standard deviation of 6.9%. Here, we found no significant correlation between participant vocabulary score and the number of words generated across each of the six VFTs. We note that there was also no difference in vocabulary performance across the experimental (exercise) and control groups (*t* < 0.38).

### 5.2. Verbal Processing

We compared the performance on the VFTs across participants who engaged in the tasks while performing vigorous exercise (experimental group) to those who were not engaged in exercise (control group). Specifically, we compared the VFT between groups across all of our lexical factors—the number of words, word length, HAL frequency, AoA, and imageability. To make these comparisons, we conducted a series of repeated-measures ANOVAs in which we used the type of test (phonemic vs. semantic) and time of test (before, during, and after) as within-participant variables, and the experiment group (exercise vs. control) as a between-groups factor. From these analyses, we find a general pattern in which the time of task impacted the performance of participants who were engaged in vigorous exercise but the performance did not change over time in participants who were not exercising.

#### 5.2.1. Lexical Factors

We recorded several properties for each word produced during the VFTs. First, we recorded number of letters, and syllables for each word. Next, we recorded the HAL frequency (Hyper Analogue of Language frequency; [37]) for each word using the English Lexicon Project online database [38]. We next noted the AoA rating for each word using several databases [39,40,41,42,43] and imageability rating for each word [43,44]. For each of these lexical factors, we conducted a repeated-measures ANOVA to examine the effect of time (before, during, and after experiment session), type of VFT (phonemic vs. semantic) and participant group (exercise or control) on the lexical characteristics of the words to examine if and how exercise influenced the type of words produced over the bout of exercise. See Table 2 for a summary of the results.

#### 5.2.2. VFT Clustering and Switching

We also compared the average cluster size and number of switches produced by each participant within each VFT. To calculate these measures, we used the methods described in detail by Troyer [14]. Participants would name as many words as they could within one minute during each VFT. They would often list a series of words that were similar in meaning (e.g., a series of arctic animals in the semantic VFT) or in sound (e.g., a series of rhyming words in the phonemic VFT). The cluster size is the sum of the words produced in sequence that share a characteristic. Switches occurred when a participant transitioned from one cluster to another. We calculated the number of switches by adding together all of the cluster transitions within a single VFT. The cluster size and switches were calculated by two separate researchers scoring each participant’s VFT output. There was a high degree of overlap (r(86) = 0.92) between raters. Once more, our analyses indicate different patterns of VFT performance between exercising and non-exercising participants.

#### 5.2.3. Number of Words

**Figure 2:**
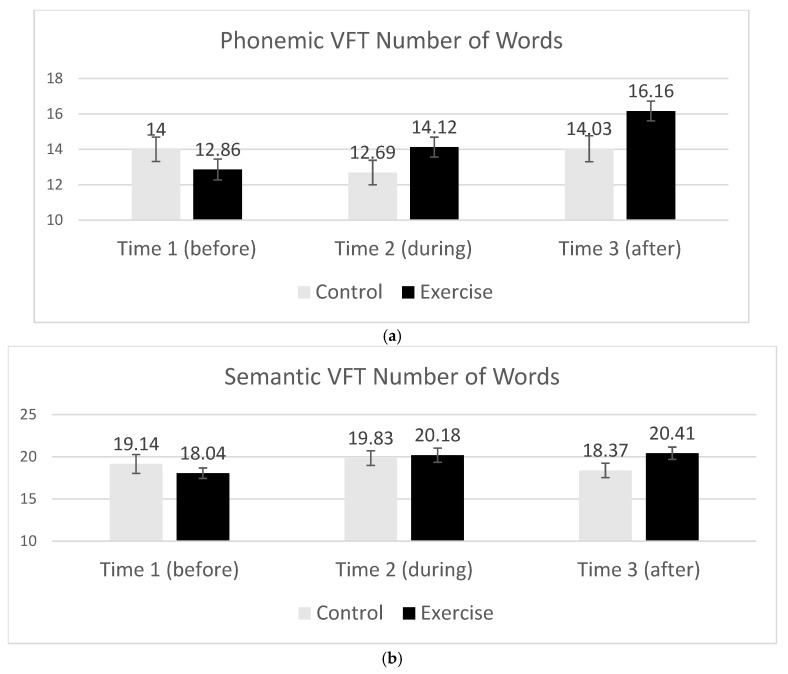
(**a**): Number of words produced in the phonemic VFT, at Time 1 (before), Time 2 (during), and Time 3 (after) for experimental (exercise) and control (non-exercise) participants. The experimental group produced more words during and after exercise compared to before exercise and compared to the control group participants. Standard error bars for each column are represented in the figure. (**b**): Number of words produced in the Semantic VFT, at Time 1 (before), Time 2 (during), and Time 3 (after) for participants in the experimental (exercise) and control (non-exercise) groups. Experimental group participants produced more words during and after exercise than before exercise and compared to the control participants. Standard error bars for each column appear in the figure.

There was no overall three-way interaction of the test type, time of test, and experiment group (F < 0.257) on the number of words produced. However, there was an interaction of the time of test and experiment group on the number of words produced (*F*(2, 164) = 4.55, *p* = 0.012, *η_p_*^2^ = 0.053) in which participants who were exercising during the VFT produced more words over time. Interestingly, exercising participants produced more words in the VFT after (M = 18.65) and during (M = 17.2) exercise compared to before exercise (M = 15.5; *t*(50) = 4.41, *p* < 0.0001; *t*(49) = 2.53, *p* = 0.015, respectively). There was a marginal difference in the number of words produced after exercise compared to during exercise (*t*(49) = 1.85, *p* = 0.07). In contrast, the participants in the control group did not change the number of words produced over time. This general pattern was the case for both the phonemic and semantic tests and is reflected in Figure 2a,b. There was also an interaction of the test type and time of test on the number of words produced (*F*(2, 164) = 3.72, *p* = 0.026, *η_p_*^2^ = 0.043) in which participants produced more words in the semantic than in the phonemic tests, overall, with this difference being most pronounced in the VFT that occurred during the middle of the session. These values can be seen in Figure 2a,b.

#### 5.2.4. Number of Syllables

There was no overall three-way interaction of the test type, time of test, and experiment group on the number of syllables produced (*F* < 0.914). However, there was an interaction of the type of test (phonemic vs. semantic) and experiment group on the number of syllables (*F*(1, 164) = 7.171, *p* = 0.009, *η_p_*^2^ = 0.08). Participants in the experimental group and control group did not differ in the number of syllables produced in the words for the phonemic tests (all *ts* < 1.39, and *ps* > 0.17). However, the experimental group produced words with more syllables than the control group across all three semantic VFTs (all *ts* > 2.056, *ps* < 0.021). The mean number of syllables produced by the experimental and control groups can be seen in Table 2. There were also the main effects of the test type and time, in which words produced in the semantic test had more syllables than in the phonemic test across time (all *ts* > 5.3, *ps* < 0.001) and participants produced words with fewer syllables across the testing session (*F*(2, 164) = 6.81, *p* = 0.001, *η_p_*^2^ = 0.077). Specifically, words with more syllables were produced after the testing session compared to before or during the testing session (*ts* > 3.53, and *ps* < 0.001). However, there was no difference in the number of syllables in responses made before compared to during the experiment session (*t* = 0.315). See Table 2 for the mean number of syllables across time and groups.

#### 5.2.5. Word Length (Number of Letters)

There was a marginal interaction of the type of test and experiment group in which the participants who were exercising produced slightly longer words in the semantic tests as compared to the phonemic tests, while this difference between tests was not as notable in the control group (*F*(2, 160) = 2.81, *p* = 0.098, *η_p_*^2^ = 0.034). There was also a main effect of the type of test in which the words produced in the semantic tests had more letters than did those produced in the phonemic tests across all time sessions (*F*(1, 160) = 129.50, *p* < 0.001, *η_p_*^2^ = 0.62, all pairwise comparisons, *ts* > 6.96, *ps* < 0.001). There was also a main effect of the length over the time of test in which the length of the words produced got shorter over time (*F*(2, 160) = 4.54, *p* = 0.014, *η_p_*^2^ = 0.052); in particular, the length of words at the end of the session were longer than those produced during or before the session (*ts* > 2.303, *ps* < 0.013), but this did not interact with experiment group.

#### 5.2.6. HAL Frequency

There was a three-way interaction of the time of test, type of test, and experiment group on the frequency of the words produced in the VFTs (*F*(2, 162) = 3.19, *p* = 0.044, *η_p_*^2^ = 0.007.). The participants in the exercise group produced lower-frequency words over time, within the phonemic task when comparing the words produced before to those produced during or after the session (*ts* > 2.612, *ps* < 0.0061) with no difference in the frequency of the words produced during compared to after the session. The exercise group did not produce words that varied in HAL frequency over time in the semantic VFT. On the other hand, the participants in the control group produced words with similar rates of word frequency across time and test (see Table 2 for the means). There also was an interaction of the time of test and experiment group on the frequency of the items produced during the VFT (*F*(2, 162) = 3.162, *p* = 0.045, *η_p_*^2^ = 0.038) such that the participants in the experimental condition were producing lower-frequency words during and after the session as compared to before the exercise session (*ts* > 2.624, *ps* < 0.0061) as they progressed through the experiment. In contrast, the control group did not produce words that differed in frequency over the course of the session (all *ts* < 0.90, *ps* > 0.188). There was also a main effect of the test type, in which participants produced lower-frequency words in the semantic as compared to the phonemic tests (*F*(1, 162) = 69.24, *p* = 0.001, *η_p_*^2^ = 0.461), across all three time points (all *ts* > 3.60, *ps* < 0.001).

#### 5.2.7. Age of Acquisition

There was no group by time interaction of the AoA of the words produced. There were consistent main effects for the type of test in which participants produced earlier acquired words in the semantic compared to phonemic tasks (*F*(1, 164) = 342.04, *p* = 0.000, *η_p_*^2^ = 0.807; all pairwise *ts* >11.28, *ps* < 0.001) and the time of test that was driven by the fact that participants produced earlier acquired words after the session as compared to during the session (*F*(2, 164) = 3.63, *p* = 0.03, *η_p_*^2^ = 0.042; *t*(84) = 3.26, *p* = 0.002)).

#### 5.2.8. Imageability

There was no interaction of the time of test with the type of test in terms of the imageability of the words that were produced by participants in both the exercise and control groups. We did find a main effect of the type of test on the imageability ratings with words produced in the semantic condition being more imageable than items produced in the phonemic condition for both the exercise and control groups (*F*(1, 98) = 832.46, *p* = 0.001, *η_p_*^2^ = 0.944; *F*(1, 168) = 1063.63, *p* = 0.001, *η_p_*^2^ = 0.927, respectively).

#### 5.2.9. Average Cluster Size

There was an interaction of the type of test and experiment group such that those participants in the exercise group were producing smaller cluster sizes than the control group, that was especially pronounced in the phonemic VFT (*F*(1, 164) = 4.711, *p* = 0.033, *η_p_*^2^ = 0.054). Control group participants were producing much larger cluster sizes (M_phon_ = 2.48 (0.43), M_sem_ = 2.93 (0.47)) than those in the experimental group (Exercise; M_phon_ = 0.363 (0.19), M_sem_= 1.15 (0.73)). In other words, the exercising participants produced smaller cluster sizes compared to the control group across the course of the session in the phonemic condition (all *ts* > 18.09, *ps* < 0.001) and in the semantic condition (all *ts* > 4.51, *ps* < 0.001). As expected, there was also a main effect of the type of test on the cluster size (*F*(1, 162) = 62.25, *p* < 0.001, *η_p_*^2^ = 0.432) in which semantic tests yielded larger cluster sizes than did phonemic tests.

#### 5.2.10. Number of Switches

**Figure 3:**
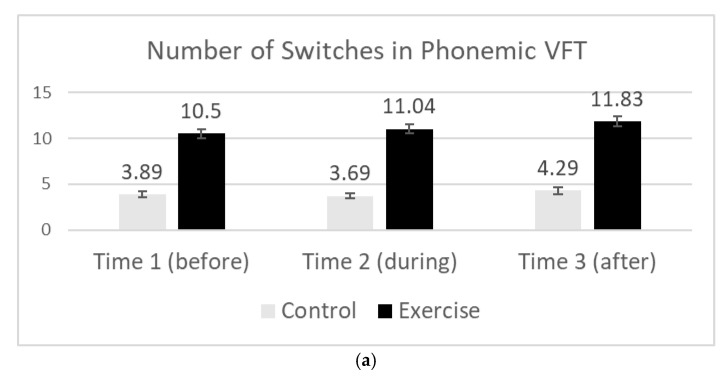

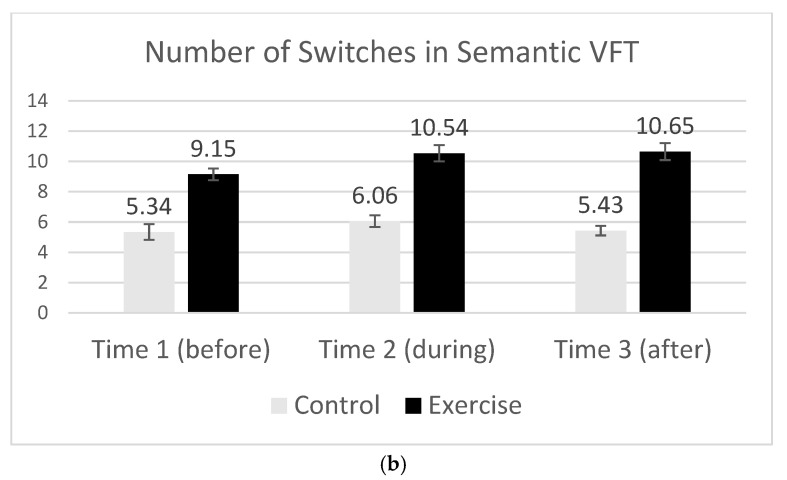
(**a**) Number of topic switches at Time 1 (before), Time 2 (during), and Time 3 (after) in the phonemic VFT. We found that the number of topic switches within the VFT increased over the course of exercise for the experimental group participants, but did not for the control group participants who were not exercising. (**b**) Number of topic switches at Time 1 (before), Time 2 (during), and Time 3 (after) in the semantic VFT. We found that the number of topic switches within the VFT increased over the course of exercise for the experimental group participants, but did not for the control group participants who were not exercising.

There was a main effect of the group in the number of switches (*F*(1, 81) = 307.33, *p* = 0.000, *η_p_*^2^ = 0.964); the exercising participants produced more switches than the control group participants (M_exercise_ = 10.7 (1.73); M_Control_ = 4.8 (1.1)). There was an interaction of the type of test and experiment such that the exercising participants produced more switches versus the control group participants, with this difference being the most pronounced in the phonemic task (*F*(1, 162) = 23.11, *p* = 0.000, *η_p_*^2^ = 0.22). That is, the difference in the number of switches between the exercise group and the control group in the phonemic condition (M_exercise_ = 11.12 (2.52); M_control_ = 3.95 (1.39); all *ts* > 11.24, *ps* < 0.001) was more pronounced than the difference in the number of switches produced in the semantic condition (M_exercise_ = 10.11 (1.91), M_control_ = 5.61 (1.47)), all *ts* > 6.16, *ps* < 0.001). See Figure 3a,b. Thus, it appears that participants completing the VFTs while exercising did not produce a large number of words associated with one another, but, instead, switched from the type of word group to another often. Overall, the number of switches increased over time for both the experimental and the control group participants (*F*(2, 162) = 3.55, *p* = 0.031, *η_p_*^2^ = 0.042), with a larger number of switches being produced after the session (M_after_ = 8.64 (3.82)) than before (M_before_ = 7.68 (3.40); *t*(82) = 3.01, *p* = 0.004) and marginally more switched during (M_during_ = 8.35 (3.81)), as compared to before the session (*t*(83) = 1.92, *p* = 0.059).

### 5.3. Executive Function Tasks

#### 5.3.1. Number Stroop

There was no three-way interaction between congruency (congruent vs. incongruent stimuli), group (exercise vs. control), and time (before or after session) on the response time in the Number Stroop task. There was an interaction of the experiment group and congruency (*F*(1, 87) = 44.41, *p* ≤ 0.001, *η_p_*^2^ = 0.34) in which the experimental group participants showed a clear RT advantage for the congruent stimuli as compared to the incongruent stimuli (*ts* > 3.9, *ps* < 0.001). The control group did not show a difference in response times for congruent compared to incongruent number Stroop stimuli before the session, but did after the session (*t*(34) = 2.43, *p* = 0.021)

#### 5.3.2. Erikson Flanker

There was no interaction between the experiment group, time of test (before vs. after), or trial type (congruent, neutral, or incongruent). There was also no interaction of the trial type and experiment group. However, there was an interaction of the time of test and experiment group such that the exercising group had shorter response times in the flanker task at the beginning of the session than did the control group (*t*(89) = 2.89, *p <* 0.005), but not after the session. There were main effects of both the time of test (*F*(1, 172) = 58.23, *p* ≤ 0.001, *η_p_*^2^ = 0.40) and trial type (*F*(2, 172) = 458.1, *p* ≤ 0.001, *η_p_*^2^ = 0.842), with shorter response times produced after as compared to before the session (*t*(87) = 7.08, *p* = *0*.001) and longer response times being produced for the incongruent compared to the congruent and neutral trials (*ts* > 23.34, *ps* < 0.001) and no difference in response times produced between the neutral and congruent trials (*t* < 1.21).

### 5.4. Results Summary

Our findings from the experimental (exercise) group of participants who were engaged in vigorous exercise indicated that they produced more words during each VFT across their bout of exercise, with the most words being produced after exercise. In addition, the lexical features of the words produced varied across the course of exercise. Participants produced lower-frequency and shorter words after exercise as compared to before exercise. This indicates that exercise was related to enhanced verbal processing in the form of greater and deeper lexical access as compared to before exercise. When we compare the performance of exercising participants to those in the control group who were not exercising, we find a general pattern in which the time of task impacted the performance of participants who were engaged in vigorous exercise but the performance did not change over time in participants who were not exercising.

## 6. General Discussion

Overall, the present study adds to the body of research indicating that exercise, especially moderate to vigorous exercise, can enhance cognitive functioning. There is support for this in the use of long-term exercise programs both to support cognitive functioning and to delay the onset of cognitive decline [1]. There is also evidence that even acute bouts of exercise can support cognitive functioning for a brief period of time after exercise [7]. This cognitive enhancement is typically illustrated in measures of executive functioning [6]. Recent evidence suggests that exercise can enhance verbal functioning, as well [11]. In the current study, we explore the positive impact that a brief bout of moderate to vigorous exercise can have on verbal processing. By using both phonemic and semantic VFTs, we see that exercise can enhance executive functioning related to the VFT and enhance lexical access. This is the first demonstration of exercise-enhanced lexical access and lexical processing.

In our comparison of VFT performance in a group of participants engaged in exercise to the VFT performance of a group of participants at rest, we can see how lexical processing is enhanced during and after exercise. In particular, we see that participants engaged in exercise produced more words, words that had more syllables (in the semantic task), and the words that they produced were of a lower-frequency (i.e., less common) across the bout of exercise. This pattern of lexical access has been associated with the enhanced processing of frontotemporal networks [19,20]. This production of more unusual words during and after exercise also suggests that cardiovascular exertion supports divergent thinking and creativity. This agrees with the results of Gondola and Tuckman [21] and Gondola [22,23] who found cardiovascular exertion enhanced creativity as measured by the “alternate uses” task (participants list as many alternate uses for common objects as possible; [47]) and the consequences task (participants brainstorm potential consequences for hypothetical scenarios, which are then coded for creativity). These two tasks are common measures of divergent thinking and creativity [48], and require the recruitment of spontaneous, flexible thinking strategies to be successful. Gondola found this increase in creativity after participants engaged in a long-term exercise program [22], as well as after engaging in a single bout of exercise [23]. In the current study, participants produced short, yet lower-frequency words after exercise as compared to before exercise. This ability to access lower-frequency words requires a deeper level of lexical processing than does accessing higher-frequency words [19,20]. From this vantage point, such deeper lexical processing is similar to the exercise-enhanced divergent thinking and creative verbal processing obtained by Gondola and colleagues.

We can examine the pattern of how the words were produced during the VFT to further demonstrate the enhanced verbal processing of the participants engaged in exercise. As people produce words in the VFT, they often group them together. For example, in the phonemic test, they often produce words such as fright, fry, and fringe, then switch to fan, fail, fake, and faint. Troyer et al. [12] referred to these as clusters and to the event in which participants move from one cluster to another as a switch. We found that our participants engaged in exercise switched topics more frequently during and after exercise compared to before exercise and more so than participants who were not engaged in exercise. Topic switching in the VFT is used in neuropsychological assessments as a measure of frontal lobe processing and executive functioning [12]. The boost in VFT switching suggests that exercise is enhancing task functions related to the frontotemporal network associated with word processing more generally [49]. We did see that the participants engaged in exercise produced smaller cluster sizes compared to participants not engaged in exercise. This clearly is related to the high number of switches that they produced during the VFT. As one switches word groupings in the VFT 9 to 12 times within a minute, the number of words that form each of those groupings cannot be very high.

The pattern of increased activation in frontotemporal networks in participants engaged in exercise (as illustrated with the enhanced lexical access and increased number of switches) aligns well with the results of fNIRS studies from Leff and colleagues [50], as well as Metzger and colleagues [11]. Specifically, Leff et al. [50] found that exercise increased blood flow to the PFC and adjacent areas including Brodman’s areas 44 and 45 (i.e., Broca’s area). Moreover, Metzger et al. [11] found that exercising while talking increased activation within these same frontotemporal areas. Once more, these are areas associated with topic switching, word choice, and depth of lexical processing [12,19,20]. Although we did not measure blood flow or electrophysiological brain activity and cannot make definitive conclusions, it is interesting to note that our pattern of VFT results coincides with predictions derived from these fNIRS studies on exercise and language processing. Our behavioral results suggest that verbal processing is influenced by cardiovascular exertion.

Surprisingly, we did not find changes in executive function performance as measured by the counting Stroop and flanker tasks. We were expecting increases in executive function performance, revealed by smaller differences in RT between congruent and incongruent trials after exercise compared to before exercise in both the counting Stroop and flanker tasks. Instead, the RT difference between incongruent and congruent trials was not changed. Possible explanations for the lack of performance change include the length of exercise and the time these tasks were administered. For example, we may have required participants to engage in vigorous exercise for too long and at too high of a level to incur improvements as measured by these executive function tasks. Previous research has indicated that the best dose of exercise to enhance cognitive function is 20–30 min [51]. In our protocol, participants exercised for 40 min (including warm-up and cool-down), and they maintained a higher intensity of exercise for 30 min compared to the participants in most of the studies cited by Verburgh et al. [8]. Cardiovascular exercise at high intensities can lead to the production of cortisol and an accumulation of lactic acid, which may hinder cognitive performance [52,53]. Our participants exercised at an average of 66% of their HRR, indicative of a vigorous exercise intensity [32], and the mean HR and RPE during the 30 min exercise bout was 154 bpm and 13.1 (e.g., perceived as ‘somewhat hard’), respectively. Therefore, if participants developed elevated levels of cortisol and lactic acid, this may have counteracted any increase in cognitive function that they would have experienced, especially in tasks relying on PFC processing. Perhaps if we had been able to measure these executive functions during exercise (as we did VFT performance) rather than just after exercise, we may have observed the expected exercise-related boost in performance. Future studies should be conducted to explore how verbal processing responses are impacted by a range of cardiorespiratory exercise. Perhaps more moderate exercise, rather than vigorous exercise, would yield both heightened verbal processing and executive function performance.

Another reason we may not have observed changes in the counting Stroop and flanker task performance could be that participants completed these tasks at the end of the protocol. After exercise, participants remained on the cycle ergometer for at least 5 min or until their HR was within 20 b·min^−1^ of their resting HR before moving from the ergometer to a desk for the final cognitive measures. They completed the last two VFTs followed by the counting Stroop and flanker tasks. Although we tried to work with participants expeditiously, any increase in executive function performance due to cardiovascular exertion may have leveled off in the intervening time.

A final reason we may not have observed increases in executive function performance across exercise may be due to the specific hemodynamic response to vigorous exercise. Metzger et al. [11] found that engaging in more challenging exercise while talking was related to specific elevated blood flow within frontotemporal areas that serve lexical processing and speech production (i.e., Brodman’s areas 44 and 45). However, this hemodynamic response is not as pronounced in adjacent areas (e.g., DLPFC) typically associated with executive functioning when a person is talking when exercising. Thus, our specific task design in which participants performed the VFT during exercise may have led to a targeted hemodynamic response that tempered the typical increase in DLPFC activation and executive function—often observed after exercise.

We did observe changes in verbal fluency behavior across exercise that likely were related to changes in neurophysiological activity associated with exercising [11]. In particular, the increases in topic switching and changes in word choices across exercise may be due to an increase in functional activity within the frontotemporal network. This is observed in the number of switches, by the increased number of words produced across exercise, and in terms of the unusualness of the words produced across exercise.

We also must acknowledge that a distinct limitation of the current study is that participants were not randomly assigned into the experimental (exercise) and control conditions. Rather, participants were asked to register for participation within an online research participation sign-up portal (Sona Systems). Each of the study conditions was listed as separate studies, one requiring two sessions (for the exercise condition) and the other requiring one session (control condition). We did pre-screen all participants based on exercise frequency, allowing only participants who were habitual exercisers (3 times a week or more for 30 min or more each time) to register for either study. Nevertheless, the lack of true random assignment leads us to be cautious when drawing strong conclusions about the differences in how the participant groups performed on the VFTs as well as our other measures.

## 7. Conclusions

In sum, it appears that cardiovascular exertion influences verbal processing. We observed increases in the number of words produced after exercise for both the phonemic and semantic VFTs. Participants also produced more unusual words as a result of exercise. These changes suggest that exercise allows more expansive thinking and deeper verbal processing. This connection between verbal processing and expansive thinking should be examined further with direct comparisons of shifts in the VFT and in measures of creativity (e.g., “alternative uses” tasks). Further, our results suggest there may be an optimal level of cardiovascular exertion for enhancing verbal performance. We could examine this further by manipulating the required HRR participants maintain while exercising and completing the VFT and other tasks. Finally, there are many ways that the current findings could be applied practically. Moderate exercise could be explored as a possible intervention for enhancing verbal processing in both narrow (e.g., military and aeronautics) and broad (e.g., students) contexts. We are excited to explore the potential ways that exercise could enhance verbal performance.

## Figures and Tables

**Table 1:** Participant Characteristics—mean values with standard deviations in paratheses. There were no significant differences between participants in the experimental (exercise) and control (non-exercise) groups except for Time 2 heart rate (*t* (87) = 52.59, *p* = 0.000).

	Experiment Group Exercise	Control Group No Exercise
Age (years)	19.33 (2.6)	19.03 (1.12)
Height (cm)	171.9 (8.2)	172.5 (8.7)
Weight (Kg)	66.0 (23.1)	70.16 (12.8)
Number of hours of weekly exercise	7.25 (3.14)	7.48 (3.14)
Resting heart rate (b·min^−1^)	65.8 (8.33)	67.23 (9.45)
Heart rate (b·min^−1^) at Time 2—during exercise (experimental) or at rest (control)	156.9 (8.1)	65.6 (7.8)
Rating of Perceived Effort (6–20 scale)	13 ± 2	N/A
Vocabulary (proportion correct in Woodcock–Johnson synonyms test)	0.732 (0.062)	0.727 (0.069)

**Table 2:** Lexical characteristics of words produced during the verbal fluency task in the experimental group (exercise). Means are reported with standard deviation in parentheses.

Measure	Type of Test	Means of Exercise Group			Means of Control Group		
		Before	During	After	Before	During	After
Number of Words	Phonemic	13.08 (4.12)	14.35 (4.02)	16.56 (4.25)	14.0 (4.10)	12.69 (4.09)	14.03 (4.33)
	Semantic	17.96 (4.35)	20.18 (5.81)	20.75 (5.28)	19.14 (6.61)	19.83 (5.17)	18.37 (5.10)
Number of Syllables	Phonemic	1.88 (0.57)	1.82 (0.511)	1.48 (0.38)	1.71 (0.48)	1.71 (0.56)	1.55 (0.43)
	Semantic	2.18 (0.38)	2.11 (0.373)	1.96 (0.33)	1.88 (0.38)	1.94 (0.40)	1.76 (0.33)
Word length	Phonemic	5.62 (0.77)	5.67 (0.82)	5.26 (0.68)	5.42 (0.74)	5.49 (0.89)	5.37 (0.83)
	Semantic	6.6 (0.87)	6.44 (0.95)	6.14 (0.76)	6.26 (0.89)	6.13 (0.94)	5.88 (0.77)
HALFreq (Means in thousands)	Phonemic	271.64 (301.0)	98.951 (177.9)	76.76 (131.6)	163.90 (301.1)	196.976 (403.8)	255.604 (498.3)
	Semantic	90.6 (39.11)	90.48 (45.58)	95.90 (33.98)	13.25 (11.32)	8.415 (42.95)	12.561 (86.5)
Age of acquisition	Phonemic	6.77 (1.14)	6.94 (1.32)	6.54 (0.94)	6.51 (1.1)	6.78 (1.14)	6.67 (0.73)
	Semantic	5.42 (0.67)	5.84 (0.53)	5.24 (0.55)	5.21 (0.56)	5.42 (0.49)	5.1
imageability	Phonemic	4.90 (0.95)	4.71 (0.57)	4.80 (0.60)	4.72 (0.60)	4.65 (0.59)	4.62 (0.55)
	Semantic	6.5 (0.27)	6.55 (0.12)	6.52 (0.14)	6.04 (0.16)	6.06 (0.14)	6.07 (0.16)

Note: We examined several lexical variables of the words produced the VFT. We were specifically interested if time of test (before, during, after) and type of test (Phonemic vs. Semantic) had similar and/or interacting effects across the lexical variables. We found the value of these lexical variables by using a number of sources including the English lexicon Project (Elp; Balota et al., 2004 [38]) as well as previously published norms of word frequency (e.g., HAL Freq, Burgess and Livesay, 1998 [37]), Age of Acquisition (AoA, Cortese & Khanna, 2007 [41], Schock, Cortese, Khanna, &Toppi, 2012 [45]) and imageability (Cortese & Fugett, 2004 [44]; Schock, Cortese, & Khanna, 2012 [43], Gao et al., 2022 [46]).

## Data Availability

The data from the present experiment are publicly available at the Open Science Framework website:https://osf.io/gv7sx/?view_only=3431b6f020d648b7bfd17e0cadf7817.

## Supplementary Materials

The following supporting information can be downloaded at: https://www.mdpi.com/article/10.3390/brainsci15010096/s1, File S1: Medical history.
