# Supplementary material for: Vigorous Exercise Enhances Verbal Fluency Performance in Healthy Young Adults
Source: Brain Sci. 2025 Jan 20;15(1):96. doi: 10.3390/brainsci15010096 (PMC11763765; doi:10.3390/brainsci15010096)
Supplement: Supplementary file 1 [file brainsci-15-00096-s001.zip › brainsci-3408095-supplementary.pdf]

Supplementary Materials

MEDICAL HISTORY

Name ______________________________ Birth Date _______________________Age _________

Local Address
____

Today's Date ______________________________ Telephone number ________________________

Email Address____

Emergency Contact/Phone Number____

Please check one for each of the questions:

1. Do you have or have you had?	Yes	When	Treatment	No
A. Diabetes mellitus	_____	_______	_______________	_____
B. Thyroid problems	_____	_______	_______________	_____
C. Hypertension (high blood pressure) _____	_______	_______________		_____
D. Epilepsy (seizures)	_____	_______	_______________	_____
E. Kidney problems	_____	_______	_______________	_____
F. Bladder problems	_____	_______	_______________	_____
G. Anemia	_____	_______	_______________	_____
H. Heart problems	_____	_______	_______________	_____
I. Coronary artery disease	_____	_______	_______________	_____
J. Lung problems	_____	_______	_______________	_____
K. High cholesterol	_____	_______	_______________	_____
L. Chest pain	_____	_______	_______________	_____
M. Back pain	_____	_______	_______________	_____
N. Gout	_____	_______	_______________	_____
O. Arthritis	_____	_______	_______________	_____
P. Chronic headaches	_____	_______	_______________	_____
Q. Dizziness	_____	_______	_______________	_____

2. Please check the following if you currently or previously have had problems in the following areas:

_____	Skin	_____	Heart	_____	Back
_____	Shoulders	_____	Blood	_____	Hips/Legs
_____	Eyes	_____	Shoulders/Arms/Hands	_____	Abdomen
_____	Ears	_____	Lungs/Chest	_____	Knees
_____	Mouth/Throat	_____	Urination/Bowel	_____	
	Arms/Hands				
_____	Ankles/Feet	_____	Nutritional	_____	Neck
_____	Weight Control	_____	Muscle Strength	_____	
	Neurologic				
_____	Mental/Emotional	_____	Genital	_____	Nose
_____	Others (specify) ____				

3. Answer the following questions:

	Yes	No
Have you ever been hospitalized as a patient?	_____	_____
Do you now have allergies or asthma?	_____	_____
Do you have any illness or injury at present?	_____	_____
Are you color blind?	_____	_____
Recent fainting/dizziness while exercising?	_____	_____
Have you ever fainted?	_____	_____
Recent head injury or loss of consciousness?	_____	_____

If you answered yes to any of the above, give details below.

4. Have any of your relatives ever had any of the following? If yes, specify which relative.

	Yes No	When	Treatment	Relative
A. Heart attack	_____ _____	_______	___________	_____
B. Thyroid problems	_____ _____	_______	___________	_____
C. Hypertension (high blood pressure)	_____ _____	_______	___________	_____
D. High cholesterol	_____ _____	_______	___________	_____
E. Diabetes	_____ _____	_______	___________	_____
F. Heart Operation	_____ _____	_______	___________	_____
G. Coronary Artery Disease	_____ _____	_______	___________	_____

5. Do you smoke

	Yes	No	Number/day
Cigarettes	_____	_____	_______
Cigars	_____	_____	_______
Pipe	_____	_____	_______

6. Do you drink alcoholic beverages? Yes _____ No _____
If yes, specify how much and how often ____

7. List all the medications/supplements you are taking: include vitamins, aspirin, etc.

Medication/Supplements	How Often	Reason
____________________	______________	_____________________________
____________________	______________	_____________________________
____________________	______________	_____________________________
____________________	______________	_____________________________
____________________	______________	_____________________________

8. Do you drink coffee? Yes _____ No _____ Number of cups/day ___________
Do you drink soda? Yes _____ No _____ Number of cups/day ___________
Do you use energy drinks? Yes _____ No _____ Number of cups/day___________

9. Do you engage in sports? Yes _____ No _____
Which ones? How often?
_________________________________ _____________________________
_________________________________ _____________________________
_________________________________ _____________________________
_________________________________ _____________________________

10. Do you experience discomfort, shortness of breath, or pain with moderate to heavy exercise?
Yes __________ No _________

If yes, please specify ____

11. List your regular physical activities:
Activity How Often How Long
__________________ _____________________ _________________________
__________________ _____________________ _________________________
__________________ _____________________ _________________________
__________________ _____________________ _________________________

I certify that the information above is correct to the best of my knowledge.

____ _________________________
Subject Signature Date

____
Study Personnel Only:
Resting Heart Rate:_______ Age: ________ Submaximal HR target: _________

Height: ________________ Weight:________________

Time of Day for Screening: _____________

Screening Sessions
15 min of cycling: measure heart rate and perceived difficulty at 1 min intervals

Heart Rate	Perceived Difficulty- 1 (easy) – 7 (very, very hard)	Watts

How many minutes after the end of cycling does it take for heart rate to reach < 100 beats per minute?
